# Improvement of L-Arabinose Fermentation by Modifying the Metabolic Pathway and Transport in *Saccharomyces cerevisiae*

**DOI:** 10.1155/2013/461204

**Published:** 2013-09-30

**Authors:** Chengqiang Wang, Yu Shen, Yanyan Zhang, Fan Suo, Jin Hou, Xiaoming Bao

**Affiliations:** The State Key Laboratory of Microbial Technology, Shandong University, Shan Da Nan Road No. 27, Jinan 250100, China

## Abstract

The L-arabinose utilization pathway was established in *Saccharomyces cerevisiae*, by expressing the codon-optimized *araA*, *araB*, and *araD* genes of *Lactobacillus plantarum*. After overexpressing the *TAL1*, *TKL1*, *RPE1*, *RKI1*, and *GAL2* genes and adaptive evolution, the L-arabinose utilization of the recombinant strain became efficient. The resulting strain displayed a maximum specific growth rate of 0.075 h^−1^, a maximum specific L-arabinose consumption rate of 0.61 g h^−1^ g^−1^ dry cell weight, and a promising ethanol yield of 0.43 g g^−1^ from L-arabinose fermentation.

## 1. Introduction

To reduce the dependence on fossil fuels, the worldwide production of bioethanol was increased from ~45 million liters in 2005 to ~113 billion liters in 2012 [1–3]. The future large-scale production of fuel ethanol will most likely be based on abundant lignocellulosic materials instead of sugar and grain, which are food for humans and animals [4]. Cost-effective fuel ethanol production from lignocellulosic materials requires the full use of the raw materials. One goal of bioethanol production is to endow the fermentation microorganism with the capacity to convert all of the sugars in lignocellulosic materials [5, 6]. Approximately 3–15% L-arabinose component can be recovered from lignocellulosic materials [7]. It is therefore necessary to construct an L-arabinose fermenting microorganism to increase the utilization of this sugar [8].

Two types of L-arabinose metabolic pathways exist in fungi and bacteria. The aldose reductase (AR), L-arabitol-4-dehydrogenase (LAD), L-xylulose reductase (LXR), and D-xylitol dehydrogenase (XDH) constitute the fungal L-arabinose metabolic pathway. The reaction catalyzed by AR and LXR is coupled with the oxidation of NADPH to NADP^+^, and the LAD and XDH use NAD^+^ as a cofactor [9]. The xylulose produced is phosphorylated and enters the pentose-phosphate pathway (PPP). The bacterial L-arabinose metabolic pathway is cofactor independent and consists of L-arabinose isomerase (AraA), L-ribulokinase (AraB), and L-ribulose-5-phosphate 4-epimerase (AraD). The D-xylulose-5-phosphate produced enters the PPP [9, 10]. Both L-arabinose metabolic pathways were established in *Saccharomyces cerevisiae*, which is the traditional ethanol-producing microorganism with excellent sugar fermenting capacity and tolerance to the harsh environment, but it cannot ferment L-arabinose [11]. Not surprisingly, a redox imbalance occurs in the recombinant *S. cerevisiae* strain containing the fungal L-arabinose metabolic pathway. The yield of the by-product L-arabitol was as high as 0.48 g g^−1^ of pentose sugar consumed in the D-xylose and L-arabinose cofermentation, although the strain expressing NADH preferred AR and LXR to decrease the redox imbalance [12].

Compared to the fungal L-arabinose metabolic pathway, the bacterial pathway is simpler and cofactor independent. However, because of the lack of effective activity assays for enzymes involved in the bacterial L-arabinose metabolic pathway, the optimization of this pathway in *S. cerevisiae *was not straightforward. The *S. cerevisiae *strain expressing the *araA*, *araB*, and *araD* genes of *Escherichia coli *could not utilize L-arabinose. However, after the *E. coli *L-arabinose isomerase gene was replaced with the *araA* cloned from *Bacillus subtilis*, the strain could grow and produce ethanol on L-arabinose after several circles of adaptive growth [13, 14]. Furthermore, the L-arabinose utilization was further improved by changing the codon usage of the bacterial *araA*, *araB*, and* araD *genes to the preferred yeast codons [15]. The L-arabinose metabolic genes of *Lactobacillus plantarum *matched the codon usage of* S. cerevisiae *more closely than the genes previously reported. Wisselink et al. [8] introduced multiple copies of *araA* and *araD* and a single copy of *araB* of *L. plantarum* into *S. cerevisiae*. After overexpressing the genes encoding the enzymes of nonoxidative PPP and extensive adaptive evolution, the resulting strain exhibited a high ethanol yield up to 0.43 g g^−1^ during anaerobic growth on L-arabinose, with a high arabinose consumption rate (0.70 g h^−1^ g^−1^ dry cell weight (DCW)) [8]. The metabolome, transcriptome, and metabolic flux analysis of a more evolved strain revealed that higher expression levels of the galactose transporter, transketolase, and transaldolase isoenzymes benefit the growth of *S. cerevisiae *on L-arabinose [16].

In the present work, the unique codon-optimized *araA*, *araB*, and *araD *genes of *L. plantarum* were expressed in the *S. cerevisiae *strain CEN.PK102-3A at different levels. Next, the genes *TAL1*, *TKL1*, *RPE1*, and *RKI1 *involved in PPP were overexpressed in this recombinant strain. The resulting strain was sequentially selected on L-arabinose under aerobic conditions and in oxygen-limited conditions. A strain with a significantly enhanced L-arabinose utilization capacity was obtained. The L-arabinose metabolic capacity of the evolved strains and the strain that also overexpressed the transporter gene *GAL2 *were investigated. The factors affecting L-arabinose metabolism efficiency are discussed.

## 2. Materials and Methods

### 2.1. Media and Culture Conditions

The yeast synthetic complete medium (SC) containing 1.7 g L^−1^ yeast nitrogen base (YNB, Sangon, China) and 5 g L^−1^ ammonium sulfate (Sangon, China), with additional carbon sources of glucose (Sangon, China) or L-arabinose (Sinopharm, China), was used for yeast cultivation. The complete supplement mixture, 0.77 g L^−1^ CSM-URA or 0.67 g L^−1^ CSM-LEU-URA (MP Biomedicals, Solon, OH), was added to maintain the required plasmids with auxotrophic selection when necessary. For strains with the *KanMX4* marker, the medium was supplied with 200 *μ*g mL^−1^ of the antibiotic G418 sulfate (Promega, Madison, WI, USA). All yeasts were cultivated at 30°C. 

### 2.2. Codon Adaptation Index Analysis

The codon adaptation index (CAI) is used to illustrate the preference of codon usage in specific species [24]. For the CAI analysis, CODONW (http://mobyle.pasteur.fr/cgi-bin/MobylePortal/portal.py?form=codonw) [15] was used. 

### 2.3. Plasmid and Strain Construction

*E. coli* DH5*α* [12] was used for subcloning. *S. cerevisiae* strains and plasmids used in this study are listed in Table 1. The primers used in this study are listed in Table 2.

**Figure 1:**
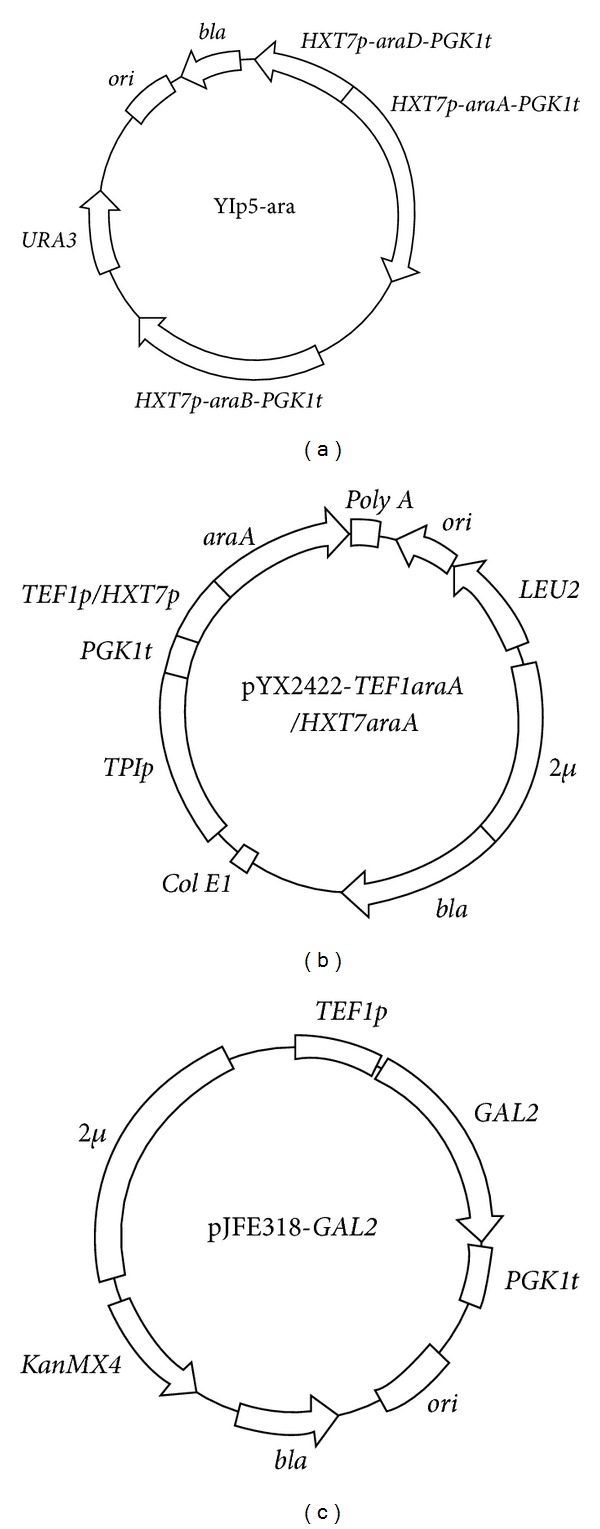
The physical maps of the plasmids (a) YIp5-ara, (b) pYX2422-*TEF1araA/HXT7araA*, and (c) pJFE318-*GAL2*.

The unique codon-optimized *araA*, *araB*, and *araD *genes encoding the L-arabinose isomerase (GenBank: CCC80517.1), L-ribulokinase (GenBank: CCC80519.1), and L-ribulose-5-phosphate 4-epimerase (GenBank: CCC80518.1) of *L. Plantarum *were artificially synthesized and ligated between the *HXT7* promoter and *PGK1* terminator sequences of plasmid pHX, which was constructed by substituting the* PGK1p* of plasmid YEp24-PGKp [20] with *HXT7p*, containing sites for the restriction enzymes *Kpn* I and *Sma* I. The *HXT7p-araD*-*PGK1t* fragment was amplified by PCR with terminal sites for the restriction enzymes *Hind* III and *Bln* I and then inserted into the *Hind* III and *Nhe* I sites of YIp5, resulting in plasmid YIp5-araD. The *HXT7p-araA*-*PGK1t* fragment containing terminal *Bgl* II and *Sal* I sites was inserted into the *Bam*H I and *Sal* I sites of YIp5-araD, resulting in the plasmid YIp5-araAD. The *HXT7p*-*araB*-*PGK1t* fragment with *Eag* I and *Stu* I sites was inserted into the *Eag* I and *Stu* I sites of YIp5-araAD, resulting in the plasmid YIp5-ara (Figure 1(a)). The *TEF1* promoter fragment (with terminal sites for *Hind *III and *Sal* I) and the *PGK1* terminator fragment (with terminal sites for *Bam*H I and *Hind *III) were cloned from the plasmids pJFE3 [23] and pYMIKP [25], respectively. These two fragments were ligated and inserted into the plasmid pYX242 to construct a vector, pYX242-WS, with two sites that can be used to express genes. Then, the gene *araA* was inserted between the *Sal* I and *Sac* I sites of this vector under control of the *TEF1* promoter and the *PloyA *terminator for its expression, and the resulting plasmid was named pYX2422-*TEF1araA *(Figure 1(b)). The plasmid pYX2422-*HXT7araA *(Figure 1(b)) was constructed using the fragment of *HXT7p* to displace the *TEF1p *fragment of plasmid pYX2422-*TEF1araA*; the joints were *Bam*H I and *Sal* I recognition sequences. The gene *GAL2 *was cloned from the chromosomal DNA of CEN.PK102-3A and then inserted into the *Xba* I and *Sal* I sites of plasmid pJFE3, resulting in plasmid pJFE3-*GAL2*. The *URA3 *fragment of plasmid pJFE3-*GAL2 *between the *Nde* I and *Apa* I sites was replaced by the *KanMX4 *gene cloned from pUG6 [18], resulting in plasmid pJFE318-*GAL2 *(Figure 1(c)).

The yeast transformation was performed using the lithium acetate transformation method [26]. The plasmid YIp5-ara was linearized at the *Stu* I site and then transformed into CEN.PK102-3A. The transformants with the *araA*, *araB*, and *araD* genes integrated into the chromosomal *URA3 *gene were selected in SC medium containing CSM-URA, and after being confirmed by sequencing, the desired transformant was named BSW1A1. Plasmids pYX242, pYX2422-*HXT7araA*, and pYX2422-*TEF1araA* were transformed into BSW1A1, resulting in BSW1AY, BSW1A7, and BSW1AT, respectively. The linearized pJPPP3, which contains the expression frames of genes *TAL1*, *TKL1*, *RPE1*, and *RKI1 *[19], was integrated into the chromosome of BSW1AT at the *GRE3* gene locus, resulting in strain BSW2AP. The strain BSW2AP was adapted on 20 g L^−1^ L-arabinose under aerobic conditions and then under oxygen-limited conditions. Once the stationary phase was reached, a new batch was initiated by transferring the culture into fresh medium with an initial biomass of 0.15 g DCW L^−1^. When the doubling time of the strain stabilized, mutant BSW3AP was selected from the adapted mutants based on its excellent growth on L-arabinose. The plasmid pJFE318-*GAL2 *was then transformed into strain BSW3AP, resulting in strain BSW3AG.

### 2.4. Real-Time Quantitative PCR

The cells were cultured in SC medium containing 20 g L^−1^ glucose and collected when the OD_600_ of cultures reached 1. The total RNA was extracted using TRIzol reagent (Sangon, China). The first strand of cDNA was reverse transcribed from 1 *μ*g of total RNA using PrimeScript RT reagent kits with gDNA Eraser (Takara, Japan). Diluted cDNA products were used for real-time quantitative PCR using the SYBR Green Real-time PCR Master Mix (TOYOBO, Japan) and the LightCycle PCR System (Roche Molecular Biochemicals, Germany). The actin-encoding gene *ACT1* was used as the reference gene for normalization. The data of real-time PCR was calculated according to the 2^−ΔΔCT^ method [19, 27]. The primers for these PCR were listed in Table 2.

### 2.5. Fermentation

A single colony was cultured overnight in SC medium containing 20 g L^−1^ glucose. A sample of the overnight culture was diluted to an initial OD_600_ of 0.5 in SC medium containing 10 g L^−1^ glucose and 10 g L^−1^ L-arabinose. After 10 h cultivation, the cells were collected and used for fermentation. All the shaker flask fermentations were performed at 30°C, 200 r min^−1^, in 200 mL shaker flasks containing 40 mL medium. The oxygen-limited condition was maintained by using a rubber stopper. The batch fermentations under anaerobic conditions were performed in 1.4 L fermentors (Infors AG, Switzerland) with a working volume of 900 mL. Anaerobic conditions were maintained by sparging with nitrogen (0.1 L min^−1^); the agitation rate was 500 r min^−1^. The pH was maintained at 5.0 by automatically pumping 1 mol L^−1^ NaOH and 1 mol L^−1^ H_3_PO_4_ [19]. The initial biomass was 0.2 g DCW L^−1^. The carbon source in the SC plus CSM-LEU-URA medium was 20 g L^−1^ L-arabinose; 200 *μ*g mL^−1^ G418 was supplied in the fermentation of strain BSW3AG. The dry cell weight of evolved strains and the unevolved strains were calculated according to the formula of dry weight (mg mL^−1^) = 0.266 × OD_600_ − 0.0762 and dry weight (mg mL^−1^) = 0.2365 × OD_600_ + 0.1149, respectively.

### 2.6. Analysis of Fermentation Products

The high performance liquid chromatography (HPLC) Prominence LC-20A (Shimadzu, Japan) equipped with the refractive index detector RID-10A (Shimadzu, Japan) was used to determine the concentrations of sugars and metabolites. The Aminex HPX-87P ion exchange column (Bio-Rad, USA) was used to analyze L-arabinose, arabitol, and ethanol at 80°C with a mobile phase of water at a flow rate of 0.6 mL min^−1^. The Aminex HPX-87H ion exchange column (Bio-Rad, Hercules, USA) was used to analyze glycerol and acetate at 45°C using 5 mmol L^−1^ H_2_SO_4_ as the mobile phase [12].

## 3. Results

### 3.1. Expression of the Codon-Optimized Genes Involved in the L-Arabinose Pathway in *S. cerevisiae*

Based on the amino acid sequence of L-arabinose isomerase (GenBank accession no. CCC80517.1), L-ribulokinase (GenBank accession no. CCC80519.1), and L-ribulose-5-phosphate 4-epimerase (GenBank accession no. CCC80518.1) recorded in the National Center for Biotechnology Information (NCBI, http://www.ncbi.nlm.nih.gov/), the *araA*, *araB*, and *araD* genes of *L. plantarum *were artificially synthesized using *S. cerevisiae *preferred codons. The CAIs of codon-optimized *araA*, *araB*, and* araD *were 0.599, 0.580, and 0.646, respectively, which were higher than those of the native sequences (0.324, 0.223, and 0.243, resp.). 

The expression cassettes of codon-optimized *araA*, *araB*, and *araD *were integrated into the chromosome of strain CEN.PK102-3A, resulting in strain BSW1A1. However, BSW1A1 could not grow on L-arabinose, although the transcribed mRNAs of these genes were all detectable. Then, more copies of *araA* were introduced into BSW1A1, carried by the episomal plasmid pYX242 and expressed under control of the *HXT7 *and *TEF1* promoters. The transcriptional levels of *araA* in the resulting strains, BSW1A7 and BSW1AT, were 12.9 ± 2.9-fold and 32.5 ± 0.7-fold higher than in the reference strain BSW1AY carrying only the integrated, expressed *araA*. The BSW1A7 and BSW1AT strains were aerobically incubated on L-arabinose, and the growth of strain BSW1AT was observed after ~150 h, whereas BSW1A7 could not grow even when cultured longer. 

### 3.2. Improvement of the L-Arabinose Utilization in *S. cerevisiae* by Engineering and Evolution

The *TAL1*, *TKL1*, *RPE1*, and *RKI1* genes involved in the nonoxidative pentose phosphate pathway were overexpressed in a single colony isolated from the BSW1AT 150 h culture by integrating the linearized plasmid pJPPP3 [19] into the chromosome. The resulting strain, BSW2AP, was evolved on L-arabinose. After 9 transfers in aerobic conditions and 12 transfers in oxygen-limited conditions, the doubling time of the culture decreased from 22 h to 4.5 h. The mutants were screened on L-arabinose plates, and a large colony was selected and named BSW3AP. 

**Figure 2:**
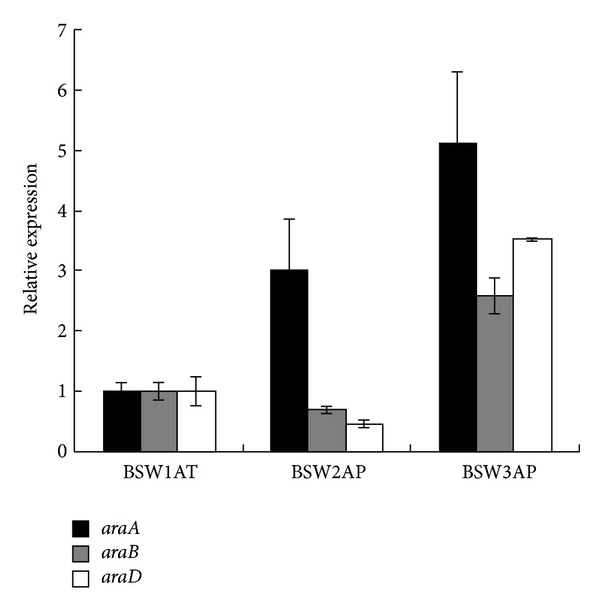
The expression of *araA* (black bars), *araB* (gray bars), and *araD* (blank bars) of strains BSW2AP and BSW3AP compared to strain BSW1AT. The fold-changes of mRNA levels of these genes are normalized to the expression of* ACT1*. The tested strains were cultivated on 20 g L^−1^ glucose. The values given are obtained from three independent measurements.

The transcriptional levels of genes in the recombinant strains BSW1AT, BSW2AP, and BSW3AP were determined by real-time quantitative PCR (Figure 2). The *araA *expression level in BSW2AP was 2-fold higher than in BSW1AT, whereas the expression levels of *araB* and *araD *in BSW2AP were lower. These changes might be due to mutations that occurred during the cultivation of BSW1AT on L-arabinose. In the evolved strain BSW3AP, all three genes were expressed at high levels. The *araA*, *araB*, and *araD* expression levels in BSW3AP were 4.1-fold, 1.6-fold, and 2.5-fold higher than those in strain BSW1AT, respectively. 

**Figure 3:**
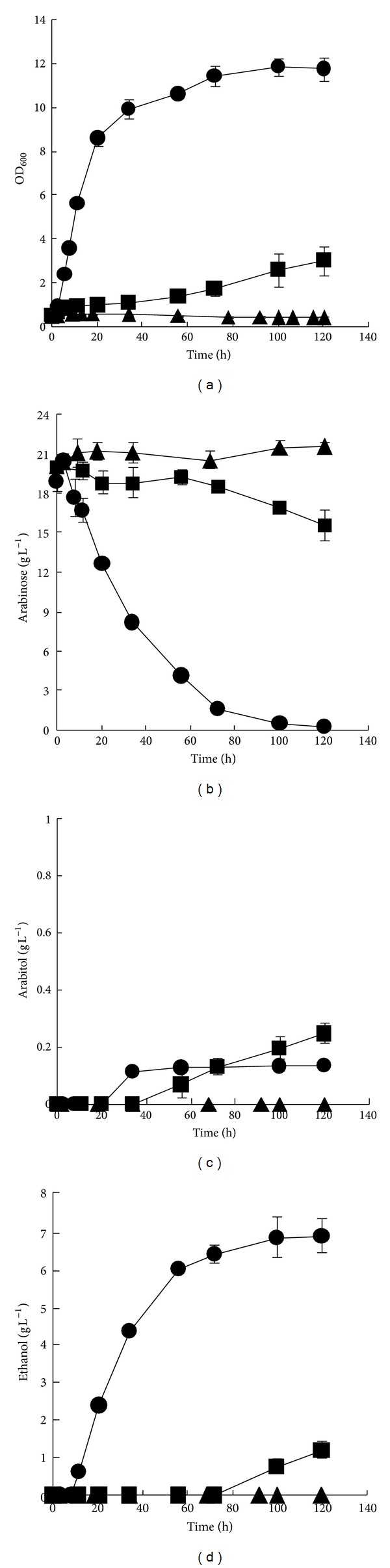
The L-arabinose fermentation of strains in shaker flasks. Growth capacity (a), L-arabinose consumption (b), arabitol formation (c), and ethanol formation (d) by BSW1AT (▲), BSW2AP (■), and BSW3AP (*⚫*). The strains were cultured in 40 mL SC medium with 20 g L^−1^ L-arabinose at 30°C, 200 r min^−1^ with an initial OD_600_ of 0.5. The data are the averages of three independent experiments.

The L-arabinose utilization of strains BSW1AT, BSW2AP, and BSW3AP was compared in shaker-flasks under oxygen-limited conditions (Figure 3); the initial OD_600_ was 0.5. No growth of BSW1AT was observed within 120 h. The strain BSW2AP grew on L-arabinose with a maximum specific growth rate (*μ*_max⁡_) of 0.011 h^−1^; 4.4 g L^−1^ L-arabinose was consumed, and 1.2 g L^−1^ ethanol was produced in 120 h of fermentation. In contrast, the *μ*_max⁡_ of the evolved strain BSW3AP increased to 0.23 h^−1^. After 120 h of fermentation, 18.6 g L^−1^ L-arabinose had been consumed with a maximum specific consumption rate of 0.7 g h^−1 ^g^−1^ DCW; 6.9 g L^−1^ ethanol had been produced, and the ethanol yield was 0.43 g g^−1^; only 0.13 g L^−1^ L-arabitol had accumulated. 

### 3.3. Overexpression of *GAL2* Improved the L-Arabinose Anaerobic Fermentation of the Evolved Strain

**Figure 4:**
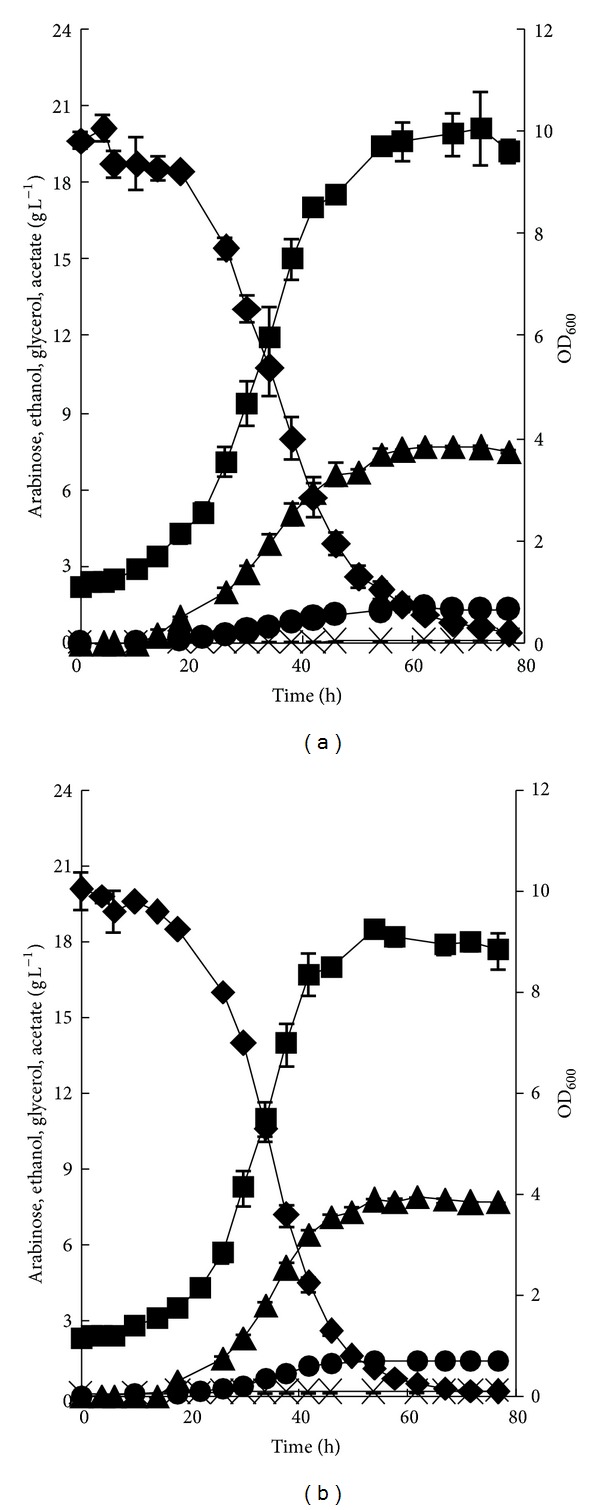
The anaerobic batch fermentation of BSW3AP (a) and BSW3AG (b) on 20 g L^−1^ arabinose. Levels of OD_600_ (■), arabinose (◆), ethanol (▲), Glycerol (*⚫*), and acetate (**×**). The fermentation was performed in 1.4 L fermentors with a working volume of 900 mL. Anaerobic conditions were maintained by sparging nitrogen (0.1 L min^−1^); the agitation rate was 500 r min^−1^. The pH was maintained at 5.0 by automatically pumping in 1 mol L^−1^ NaOH and 1 mol L^−1^ H_3_PO_4_. The initial biomass was 0. 2 g DCW L^−1^. The 20 g L^−1^ L-arabinose was used as the carbon source in SC plus CSM-LEU-URA medium, and 200 *μ*g mL^−1^ G418 was supplied in the fermentation of strain BSW3AG. The data are the average of duplicate determinations.

The galactose permease gene *GAL2 *was overexpressed in BSW3AP, resulting in strain BSW3AG. The anaerobic L-arabinose fermentation properties of strain BSW3AP and BSW3AG were studied (Figure 4 and Table 3) in bioreactors. Strain BSW3AP grew on L-arabinose with a maximum specific growth rate of 0.067 h^−1^. The maximum specific consumption rate of L-arabinose was 0.49 g h^−1 ^g^−1^ DCW. Ethanol was produced at a maximum specific rate of 0.20 g h^−1 ^g^−1^ DCW with a yield of 0.42 g g^−1^. The overexpression of *GAL2 *significantly improved the L-arabinose fermentation capacity. The maximum specific growth rate of BSW3AG was 0.075 h^−1^, which was 12% faster than that of BSW3AP. The L-arabinose specific consumption rate of BSW3AG was 0.61 g h^−1 ^g^−1^ DCW, which was 24% faster than that of BSW3AP. The ethanol production rate was 0.27 g h^−1 ^g^−1^ DCW, and the ethanol yield was 0.43 g g^−1^. Furthermore, both BSW3AP and BSW3AG produced small amounts of glycerol (1.4 g L^−1^ for both strains) and almost undetectable amounts of arabitol and acetate. 

## 4. Discussion

The complete conversion of sugars is important for efficient and cost-effective fuel ethanol production from lignocellulosic materials. Even small improvements in substrate utilization can significantly decrease the costs of the whole process [28]. L-arabinose is an important component of lignocellulosic materials. Expression of the *L. plantarum* L-arabinose pathway has proven to be effective in constructing L-arabinose utilizing *S. cerevisiae *[8]. Given that the codon-optimized genes might lead to increased expression of the proteins [15, 29], in the present work, the original *araA*, *araB*, and *araD* genes of *L. plantarum* were modified to match the codon usage of *S. cerevisiae *and then integrated into the chromosome of strain CEN.PK102-3A. However, this recombinant strain could not grow on L-arabinose. More copies of the *araA* gene were then introduced into the recombinant strain under the control of the *HXT7 *and *TEF1* promoters. When the two resulting strains were cultured on L-arabinose, growth was only observed in cultures of the strain expressing* araA *under the control of the *TEF1 *promoter, in which the *araA *transcriptional level was 1.4-fold higher than in the strain expressing* araA *controlled by the *HXT7 *promoter. We suggest that only when the transcription level of *araA *is higher than a certain level can growth on L-arabinose occur. In contrast, only one copy of *araB* and *araD *was introduced into this recombinant strain, and the transcriptional levels of these genes were lower than in the parental strain. These phenomena indicated that *araB* and *araD* were less important for growth on L-arabinose because only one copy of these genes allowed the recombinant strain to grow on L-arabinose. 

Adaptive evolution was proven to be a powerful method to enhance the strains' metabolic efficiency [8, 13]. In the present study, the evolved strain BSW3AP shows significantly improved L-arabinose metabolizing capacity. The increased transcription levels of all the three genes (*araA*, *araB*, and *araD*) might contribute to the enhancement. Compared to *araA* and *araD*, the expression level of *araB *was lower. Becker and Boles [13] reported that a mutant on L-arabinose decreased the L-ribulokinase activity expressed by *araB*. The relatively lower expression of *araB *avoids the overconsumption of ATP, which would benefit the growth of the strain on L-arabinose. 

L-arabinose is a novel carbon source for *S. cerevisiae*. The uptake of L-arabinose in *S. cerevisiae* mainly depends on the nonspecific transport by the hexose transporter Gal2p. The Hxt9p and Hxt10p also can transport L-arabinose, but the efficiency is very low [30]. It was reported that overexpressing *GAL2 *improves the L-arabinose utilization [13, 16]. In this study, overexpressing *GAL2 *notably increased the growth rate and L-arabinose consumption rate of our evolved strain BSW3AP. This result suggested that the theoretical L-arabinose metabolic flux was higher than we detected in BSW3AP. The L-arabinose utilization of BSW3AP was limited by its absorption rate. When the *GAL2* was overexpressed, more Gal2p in the plasma membrane lead to an increased L-arabinose uptake and then promote the L-arabinose utilization. Our result further confirmed the importance of transporters for L-arabinose utilization; however, the affinity of Gal2p for L-arabinose is low, and glucose competitively inhibited its binding to L-arabinose [30]. Improving the efficiency of the L-arabinose specific transporter remains to be conducted.

## 5. Conclusions

With multiple steps of genetic engineering and adaptive evolution, we obtained the strain BSW3AG, which grows on L-arabinose with a *μ*_max⁡_ of 0.075 h^−1^. The maximum specific L-arabinose consumption rate is 0.27 g h^−1^ g^−1^ DCW, and the maximum ethanol yield is 0.43 g g^−1^ L-arabinose consumed, which is 84.3% of the theoretical amount. A high level of *araA* expression is notably important in establishing an efficient L-arabinose pathway in *S. cerevisiae*, and more efficient transporters are necessary to improve the L-arabinose absorption capacity of the evolved strains. 

## Figures and Tables

**Table 1:** *S. cerevisiae* strains and plasmids used in this study.

	Relevant genotype	Source/reference
Strain		
CEN.PK102-3A	*MAT*α* leu2-3*,* 112 ura3-52 *	[17]
BSW1A1	CEN.PK102-3A derivative; {YIp5-ara}	This work
BSW1AY	CEN.PK102-3A derivative; {YIp5-ara, pYX242}	This work
BSW1A7	CEN.PK102-3A derivative; {YIp5-ara, pYX2422-*HXT7araA*}	This work
BSW1AT	CEN.PK102-3A derivative; {YIp5-ara, pYX2422-*TEF1araA*}	This work
BSW2AP	BSW1AT, *gre3 (−241, +338):: TPI1p-RKI1-RKI1t-PGK1p-TAL1-TAL1t-FBA1p-TKL1-TKL1t-ADH1p-RPE1-RPE1t-loxP *	This work
BSW3AP	BSW2AP, selected for oxygen-limited growth on L-arabinose	This work
BSW3AG	BSW3AP derivative; {pJFE318-*GAL2*}	This work
Plasmid		
pUG6	*E. coli* plasmid with segment *LoxP-KanMX4-LoxP *	[18]
pJPPP3	pUC19-based yeast integration plasmid, containing *GRE3*-targeting recombinant arms, overexpression cassette of *Sc-TAL1*, *Sc-TKL1*, *Sc-RPE1*, *Sc-RKI1*, and selectable marker *loxP-KanMX4-loxP *	[19]
YEp24-PGKp	2*μURA3 *	[20]
pHX	YEp24-PGKp *PGK1p::HXT7p *	This work
YIp5	Integration plasmid, *Ura3 *	[21]
YIp5-ara	YIp5-*HXT7p*-*araA*-*PGK1t*-*HXT7p*-*araB*-*PGK1t*-*HXT7p*-*araD*-*PGK1t*, and selectable marker *loxP-KanMX4-loxP *	This work
pYX242	2*μLEU2 *	[22]
pYX242-WS	pYX242-*PGK1t*-*TEF1p *	This work
pYX2422-*TEF1araA *	pYX242-*PGK1t*-*TEF1p*-*araA *	This work
pYX2422-*HXT7araA *	pYX242-*PGK1t*-*HXT7p*-*araA *	This work
pJFE3	2*μURA3 *	[23]
pJFE3-*GAL2 *	*pJFE3*-*TEF1p*-*GAL2*-*PGK1t *	This work
pJFE318-*GAL2 *	pJFE3-*GAL2 URA3::KanMX4 *	This work

**Table 2:** Oligonucleotides used in this work.

Primers	Sequence (5′-3′)	Purpose
Hxt7 upstream-HX	CATAGATCTCTCACAAATTAGAGCTTCAATTTAAT	Cloning the fragment of *HXT7p*-*araA*-*PGKt1 *
Pgk6 downstream-S	CATGTCGACAGCAATTTAACTGTGATAAACTACCG	Cloning the fragment of *HXT7p*-*araA*-*PGKt1 *
Hxt7 upstream-EEB	CATCGGCCGAGATCTCCTAGGCTCACAAATTAGAGCTTCAATTTAAT	Cloning the fragment of *HXT7p*-*araB*-*PGKt1 *
Pgk6 downstream-S	CATGTCGACAGCAATTTAACTGTGATAAACTACCG	Cloning the fragment of *HXT7p*-*araB*-*PGKt1 *
Hxt7 upstream	CATCCTAGGCTCACAAATTAGAGCTTCAATTTAAT	Cloning the fragment of *HXT7p*-*araD*-*PGKt1 *
Pgk6 downstream	CATCCTAGGAGCAATTTAACTGTGATAAACTACCG	Cloning the fragment of *HXT7p*-*araD*-*PGKt1 *
HXT7p-F	CCCAAGCTTCTCACAAATTAGAGCTTCAATT	Cloning *HXT7p *
HXT7p-R	ACGCGTCGACATTGGATCTAGATGCATTCGCG	Cloning *HXT7p *
TEF1 W up	CCCAAGCTTCACAATGCATACTTTGTACGTT	Cloning *TEF1p *
TEF1 W down	GCGCGTCGACTTGTAATTAAAACTTAGATTAG	Cloning *TEF1p *
AraA W up	ACGCGTCGACATGTTATCTGTTCCTGATTATG	Cloning *araA *
AraA W down-His	TACGAGTCTTTAGTGGTGGTGGTGGTGGTGTTTTAAAAATGCTTTTGTCA	Cloning *araA *
AraA-F	CAAGCAGGTGGTGGTCATCATAC	For quantitative real-time PCR of *araA *
AraA-R	TACCAACCATTGTAGCGTAATCTTCC	For quantitative real-time PCR of *araA *
AraB-1F	ATGCAGCATTCGCACCTTTG	For quantitative real-time PCR of *araB *
AraB-1R	CCTTCACCTGCTGTGGACAT	For quantitative real-time PCR of *araB *
AraD-1F	CCAGCTGCAGATGCATTAACT	For quantitative real-time PCR of *araD *
AraD-1R	ACAGCCTTAGCTGGTGTTGG	For quantitative real-time PCR of *araD *
Gal2 up	GCTCTAGAATGGCAGTTGAGGAGAACAATATGC	Cloning *GAL2 *
Gal2 down	ACGCGTCGACTTATTCTAGCATGGCCTTGTAC	Cloning *GAL2 *
pG418-Apa I up	AGTGGGCCCTAGGTCTAGAGATCTGTTTAGC	Cloning *KanMX4 *
pG418-Nde I down	GGAATTCCATATGATTAAGGGTTCTCGAGAGCTCG	Cloning *KanMX4 *

**Table 3:** The maximum specific growth rates (*μ*_max⁡_), the maximum specific L-arabinose-consumption rate, the ethanol production rate, and the ethanol yield for BSW3AP and BSW3AG on 20 g L^−1^ L-arabinose.

Strain	*μ*_ max_ (h^−1^)	The maximum specific L-arabinose consumption rate (g h^−1^ g^−1^ DCW)	Ethanol production rate (g h^−1^ g^−1^ DCW)	Ethanol yield (g g^−1^ L-arabinose consumed)
BSW3AP	0.067	0.49	0.20	0.42
BSW3AG	0.075	0.61	0.27	0.43
